# Evaluation of the antihyperglycemic and antihyperlipidemic effects of *Trigonella foenum-graecum* L and *Coffea arabica* L seeds in STZ induced diabetic mice: impact on kidney and liver functions

**DOI:** 10.11604/pamj.2024.49.94.44735

**Published:** 2024-11-27

**Authors:** Daniel Muluye, Paulos Getachew, Tiwabwork Tekalign, Samuel Woldekidan, Tesfaye Biftu

**Affiliations:** 1 Department of Clinical Nutrition, Centre for Food Science and Nutrition, Addis Ababa University, Addis Ababa, Ethiopia,; 2 Department of Nursing, School of Nursing, College of Medicine, and Health Science, Arba Minch University, Arbaminch, Ethiopia,; 3 Armauer Hansen Research Institute, Traditional and Modern Medicine Research and Development Directorate, Addis Ababa, Ethiopia,; 4 Institute of Pharmaceutical Sciences, Adama Science and Technology University, Adama, Ethiopia

**Keywords:** Diabetes mellitus, galactomannan-treated, chlorogenic acid-treated, hyperglycemia, hyperlipidemia, streptozotocin

## Abstract

**Introduction:**

diabetes mellitus is a metabolic disease of the endocrine system characterized by elevated blood sugar levels due to disorders in insulin action, and secretion. This study aims to evaluate the antihyperglycemic and antihyperlipidemic effects of Trigonella foenum-graecum L and Coffea arabica L seeds in STZ (streptozotocin) induced diabetic mice: impact on kidney and liver functions.

**Methods:**

twenty-six male mice aged 2 weeks, were divided into five groups: normal control, diabetic control (DC), positive control (PC), galactomannan-treated (GM), and chlorogenic acid-treated (CGA). Trigonella foenum-graecum L (TFL) seeds were bought from the local market. Similarly, Coffea arabica L (CAL) seeds were collected from the Ethiopian Commodity Exchange. After a 28-day treatment period TFL and CAL, Fasting Blood Glucose diabetic control (FBG], Oral Glucose Tolerance (OGT), lipid profile, and kidney and liver function tests were conducted. Statistical analysis was performed using R software, with the significance level set at a P-value < 0.05.

**Results:**

galactomannan-treated and CGA significantly reduced FBG by -176.8 and -252.4 (p<0.01), respectively, and improved OGT (AUC of CGA-7449 and GM-14754) in comparison to DC (52452] (p<0.05). Similarly, liver and kidney function tests showed a statistically significant difference against the non-treated group 1 (p<0.01). In the lipid profile test, galactomannan reduced triglyceride and cholesterol levels, while chlorogenic acid improved low-density lipoprotein and cholesterol.

**Conclusion:**

according to this research, TFL and CAL seeds decreased fasting blood glucose levels and improved glucose tolerance, lipid profiles, liver and kidney function tests, and glucose tolerance.

## Introduction

Diabetes mellitus is a group of metabolic disorders with the main feature of chronic hyperglycemia. It is either impaired insulin secretion or insulin resistance, or usually both [1]. The global incidence and prevalence of type 1 diabetes is increasing. It is estimated that 1 in 11 adults has diabetes mellitus (90% have type 2 diabetes mellitus (T2DM)) worldwide. Type 2 diabetes mellitus and its complications have reached epidemic levels, particularly in developing countries [2]. In the years to come, the largest increase will take place in regions of low and middle income as a consequence of population ageing, growth, and urbanization [3]. In these countries, insulin will be difficult to obtain and afford. Cost-effective therapeutic and preventive strategies shall be implemented [4]. Research into potential nutraceuticals is both essential and necessary [5]. *Coffea arabica* L (green coffee) beans reported to contain many polyphenolic compounds, which give them antioxidant properties [6], nutraceuticals that enhance insulin sensitivity, and hepatoprotective benefits [7]. One of the nutraceuticals in coffee, chlorogenic acid (CGA), has been suggested for the management of body weight [8], prevention, and treatment of chronic diseases [9], including type II diabetes [10]. Moreover, its potential to reverse fasting blood glucose (FBG) has been reported [11,12].

Whereas, insignificant effects on blood glucose concentration are also reported [13]. It decreases serum liver biomarkers as well as markers of oxidation, inflammation, and apoptosis in rats [14]. Moreover, CGA has been reported to reduce lipogenesis and increase fatty acid β-oxidation [15], which could be the reason for its anti-obesity effect [8]. *Trigonella foenum-graecum* (fenugreek) is a medicinal herb [16] with bioactive molecules such as diosgenin, 4-hydroxyisoleucine, furostanolic saponins, and fiber (GM). Of which GM has been reported for the majority of fenugreek´s positive effects against diabetes [17]. Besides the experimental findings, molecular-dynamic studies suggested that it could be a viable therapeutic candidate for type 2 diabetes [18]. Moreover, it has demonstrated remarkable efficacy in both preventing and treating diabetic nephropathy, a serious complication of diabetes [19]. Potent hypoglycemic (blood sugar-lowering) properties, coupled with its robust antioxidant activity, enabled GM to significantly alleviate liver and kidney damage induced by streptozotocin (STZ), a chemical used to induce diabetes in experimental models. Furthermore, GM has been consistently shown to be highly effective in managing hyperglycemia (high blood sugar levels) in various studies [20,21]. It's been found to be an effective agent for hypolipidemic or lipid-lowering medicine. Therefore, it has the potential to be a useful lipid-lowering herb. Control of glycemia, lipid profile, and liver and kidney function tests are investigated in a scattered manner. The necessity of further studies warranted a vast number of reviews and studies. chlorogenic acid [6-9] and GM [22-25] are some of the research recommendations considering one or more of the preceding factors. The aim of this study was to investigate the impact of CGA and GM on fasting blood glucose, glucose tolerance, lipid profile, as well as liver and kidney function tests.

## Methods

**Figure 1:**
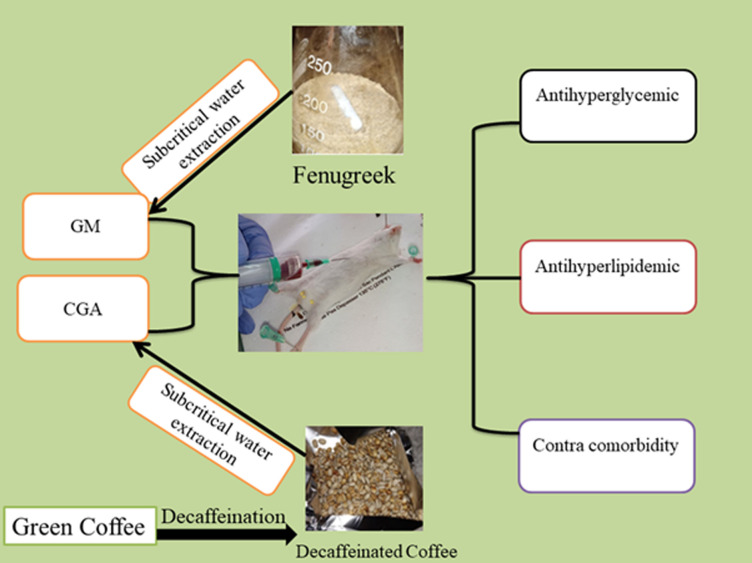
graphical abstract: effects of subcritical water extractions of *Trigonella foenum-graecum* L and *Coffea arabica* L seed in STZ induced diabetic mice: impact on kidney and liver functions

**Study design:** the study was conducted from September to December 2021 in the Ethiopian Public Health Institute (EPHI) and Addis Ababa University Center of Food Science and Nutrition Laboratories. The mice were divided into five groups. Group 1: normal control, non-diabetic control; group 2 (DC): diabetic control, administered tap water only; group 3 (PC): positive control, diabetic, treated with 5 mg/kg of glibenclamide; group 4 (CGA): diabetic treated with 100 mg/kg of chlorogenic acid; and group 5 (GM): diabetic treated with 100 mg/kg of galactomannan. The fasting blood glucose level (FBG), lipid profile, kidney, and liver function tests and oral glucose tolerance test were tested. Mice in all groups were administered once daily for 28 days. Fasting blood glucose level was measured by draining blood from the tail of each mouse (Figure 1) [19].

**Materials and reagents:** *Trigonella foenum-graecum* (fenugreek) seeds were bought from local market, Gondar, Ethiopia. Similarly, *Coffea arabica* L (green coffee) seeds were collected from the Ethiopian Commodity Exchange (ECX) coffee warehouse, in Addis Ababa, Ethiopia. The collected samples were rinsed and dried to remove any foreign debris. Hundred (100g) of each the samples were weighed and ground into a fine powder using a blender for further use. Streptozotocin was purchased from Sigma Aldrich (St. Louis, MO, USA), glucometer, glibenclamide, sodium chloride, glucose powder, and oral gavage (needles and syringe) were used to perform the biological activity evaluation.

### Preparation of seed extracts

**Figure 2:**
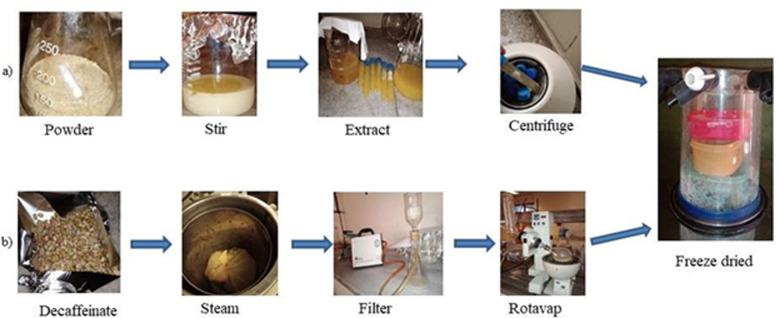
schematic representation of the extraction procedure of galactomannan (GM) (a) and chlorogenic acid-treated (CGA) (b)

**Galactomannan extraction:** the extraction of GM was prepared by grinding fenugreek seed (0.5 mm mesh) and dispersed in distilled water at a ratio of 1: 40 (W/V) for 4h. After magnetically stirring under hot water for an hour, the supernatant was centrifuged at 17,700 x g for 10 minutes. Then, the supernatant was mixed with absolute ethanol at a ratio of 1: 1 (v/v) to precipitate the dietary soluble fiber, GM. Finally, freeze-dried to remove the remaining wet content, and GM powder was collected and stored in a desiccator (Figure 2) [24].

**Decaffeination and chlorogenic acid extraction:** the decaffeination of dried green coffee was conducted according to previously reported work [26] with minor modifications. Briefly, the dried green coffee seeds were soaked in 80% ethanol for 1 hour. Then, the ethanol was removed using a rotatory evaporator. Then, the beans get steamed to remove the solvent left on the seed. Next, the seed was dried before being grinded into fine powder for CGA extraction. A subcritical water extraction method was applied for CGA extraction. Green coffee seed powder dispersed in distilled water at a ratio of 1: 20 was magnetically stirred at 800c for 1 hour. Then the supernatant was filtered using *Whatman no. 1* filter paper. The final wet product was lyophilized to get the CGA powder (Figure 1).

**Animals and ethics statement:** twenty-six male mice (20 - 30g) were obtained from Ethiopian Public Health (EPHI) animal breeding unit, in Ethiopia. The animals were treated in accordance with Organisation for Economic Co-operation and Development (OECD) guidelines [27]. Accordingly, they were acclimatized for 5 days. All the experimental mice were kept in an environmentally controlled room (temperature: 22 ± 3°C, humidity: 60 ± 10%, and 12 h light/dark cycle). All the mice had free access to tap water and unrestricted pellet access.

**Induction of diabetes:** in this study, streptozotocin (STZ) was used to induce diabetes. Fresh sodium citrate buffer (pH= 4.5) was used to dissolve the STZ. A single high dose (150 mg/kg) of STZ had been injected intraperitoneally to induce hyperglycemia. After 72 hours of induction, animals fasted for 3-4 hours and the level of their FBG was measured. Only mice with FBG levels > 250 mg/dL were considered diabetic [28].

**Acute toxicity study:** the OECD guidelines number 423 (up and down procedure) was referred to determine acute oral toxicity of the aqueous extract of *Trigonella foenum-graecum* L. and *Coffea arabica* L. A starting dose of 2000mg/kg, B.W was administered to three albino female mice orally and observed for 14 days [27].

**Fasting blood glucose and oral glucose tolerance test:** the FBG test was conducted immediately after fasting and before feeding glucose for OGTT examination. Animals were fed respective diets for 28 days with oral gavage. Following 3-4 hours of fasting, each mice received glucose 3 g/kg/body weight. Then, blood glucose was taken from the tail tip at 0, 30-, 60-, 90-, and 120 minutes using a glucometer (One Basic, Inc.).

**Blood collection and biochemical analysis:** at the end of the experiment, the animals were anesthetized and blood samples were collected into a serum jell tube after 3-4 hours of fasting. Serum was prepared by centrifugation at 3000 rpm for 10 minutes The serum was kept at a temperature of -80°C for biochemical analysis. Clinical biochemistry such as aspartate aminotransferase (AST), alanine aminotransferase (ALT), alkaline phosphatase [29], bilirubin (BIL), urea and creatinine levels were evaluated to determine the liver and kidney functions of experimental animal [30].

**Determination of lipid profile:** all cholesterol, high-density lipoprotein (HDL), low-density lipoprotein (LDL), triacylglycerol (TAG), and total cholesterol (TC) [24].

**Sample size determination and randomization:** the OECD guidelines on the number of animals were considered and met [27]. The induced mice were randomly allocated to either of the treatment or control groups. Each coded mouse was labeled with a Roman number from I to V. After making a table with 5 rows and 4 columns, the first group was randomly distributed to the four groups under intervention. Then, the remaining mice in a cage were relocated to the four groups, following their similar code.

**Ethical approval:** it was sought and obtained from the College of Natural and Computational Sciences Institutional Review Board (AAUCNCSIRB), Addis Ababa University, Ethiopia (Ref No. CNSDO 407 12 2020) before the commencement of animal studies. The methods utilized in this study adhere to the EPHI laboratory and OECD guidelines.

**Data analysis:** the data were analyzed using the R statistical software package and Prism Graph Pad v.20. Differences in group mean and which group differs were observed by applying two-way ANOVA and post-hoc Bonferroni test, respectively. P-values of < 0.05 were considered statistically significant. The results were expressed as mean ± standard deviation unless otherwise stated.

## Results

**Acute toxicity:** the aqueous extracts of *Trigonella foenum-graecum* L and *Coffea arabica* L were safe up to a dose of 2 g/kg/body weight and produced neither mortality nor any indications of clinical complications in the treated experimental animals during the 14-day observation period after administration of the plant material.

**Figure 3:**
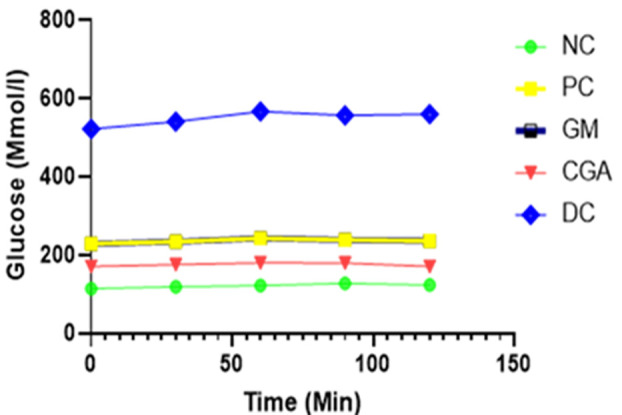
the effect of CGA and GM on oral glucose tolerance; prism graphpad AUC analysis of OGTT post 0 to 120 minutes; key: NC: normal control; DC: diabetic control; PC: positive control; GM: galactomannan; CGA: chlorogenic acid; AUC: area under the curve

**Effects of chlorogenic acid (CGA) and galactomannan (GM) on FBG and OGTT:** the FBG was tested before and after 28 days of treatment The results obtained are presented in Table 1. The results demonstrated that the initial FBG level of the mice was similar among all STZ-induced groups. Significant mean difference (p< 0.05) was observed in both treatments as indicated in Table 1. Whereas, in the post-test, both treatment groups showed significant mean differences with p-value of ≤ 0.01. Chlorogenic acid and GM at 100 mg/kg/day for 28 days lowered fasting blood glucose levels (P=1.32e-05 and 0.000131, respectively) compared to the diabetic control group (Table 2). After 4 weeks of treatment, Wider AUC ((μg/mL)•min) is recorded for DC (52452) and a lesser for CGA (7449), followed by GM (14754) as summarized in Figure 3 and Table 3. Galactomannan also showed a comparable AUC with PC. Hence, both extracts boosted glucose tolerance with the weight exceeded by CGA.

**Table 1:** the effect of chlorogenic acid-treated and galactomannan-treated on fasting blood glucose in mice; mean difference between pre and post tests

Treatment	Pre	Post	mean Diff	Median	
NC	129.6+33.54	114.4+12.64	*-15.2	127	120
DC	388.8+62.12	522.2+23.95	133.4	402	528
PC	377.8+49.68	144.5+15.01	***-233.3	380	144.5
GM	405.8+65.31	229+34.58	**-176.8	410	227
CGA	423.4+72.51	171+22.37	***-252.4	410	170

Significance codes 0 ‘***’ 0.001 ‘**’ 0.01 ‘*’ 0.05 ‘*.’ **Note**: The mean difference is significant at p<0.05 as compared to DC Data are expressed as Mean + SD, n=5, NC=6. NC: normal control, DC: diabetic control, PC: positive control, GM: galactomannan and CGA: chlorogenic acid

**Table 2:** the relative impact of chlorogenic acid-treated and galactomannan-treated on FBG against diabetic control group

	Time	Y	Group 1	Group 2	P	P adj	P adj signif
15	2	FBG	2	3	6.85e-07	6.85e-06	****
16	2	FBG	2	4	0.000131	0.001	**
17	2	FBG	2	5	1.32e-05	0.000132	***

Bonferroni test between treatment Groups against DC. Group 2 refers to DC while 3, 4, and 5 represent PC, GM, and CGA respectively. y- outcome variable, p<0.05, p.adj [p adjusted for Bonferroni correction] NC: normal control; DC: diabetic control; PC: positive control; GM: galactomannan and CG: chlorogenic acid

**Table 3:** the effect of chlorogenic acid-treated and galactomannan-treated on oral glucose tolerance, AUC as a reference

	NC	PC	GM	CGA	
Baseline	114.4	114.4	114.4	114.4	114.4
Total Area ± Std. Error	891 ± 922.5	14754 ± 1045	14754 ± 1190	7449 ± 930.8	52452 ± 1090
95% Confidence Interval	0.000 to 2699	12705 to 16803	12422 to 17086	5625 to 9273	50316 to 54588

Effect of CGA and GM on oral glucose tolerance test [OGTT]; area under the curve (1) for; Area ± Std. Error; GM [14754 ± 1190]; CGA [7449 ± 930.8], both are significant [p <0.05] in comparison to DC [52452 ± 1090]. NC: normal control; DC: diabetic control; PC: positive control; GM: galactomannan and CGA; chlorogenic acid, AUC: area under the curve

**Effect of chlorogenic acid and galactomannan on liver and kidney function:** in the liver function test, all the parameters measured (ALT, AST, ALP, and bilirubin) from the GM group showed a significant difference against DC. For CGA, AST was insignificant, and ALP showed a less significant level compared to GM. Whereas, kidney function tests (creatinine and urea) disclosed a comparable effect against DC (P< 0.00) (Table 4).

**Table 4:** liver and kidney function tests among streptozotocin-induced diabetic mice

Groups	ALT [u/l]	AST [u/l]	ALP [u/l]	Bilirubin	Urea [mg/dl]	Creatinine [mg/dl]
**NC**	***31.2 **±** 6.90	***103 **±** 46.5	***58.2 **±** 8.84	***0.21 **± 0.15**	***38.4 **±** 6.13	***0.14 **±** 0.05
**DC**	396 **±** 172	550 **±** 69.70	185 **±** 56.90	0.62 **± 0.06**	119 **± 23.80**	0.86 **±** 0.06
**PC**	***73.3 **±** 9.38	**284 **±** 45.80	**89.7 **±** 5.13	***0.20 **± 0.08**	***48.4 **±** 4.23	***0.26 **±** 0.02
**GM**	***41.6 **±** 11	***195 **±** 64	***74.8 **±** 21	***0.10 **± 0.06**	***44.8 **± 9.40**	***0.19 **±** 0.05
**CGA**	***54.2 **±** 5.80	464 **±** 128	**85.2 **±** 23.50	***0.11 **± 0.03**	***64 **± 9.64**	***0.24 **±** 0.08

Significance codes 0 ‘***’ 0.001 ‘**’ 0.01 ‘*’ 0.05 ‘*.’ **Note**: The mean difference is significant at p<0.05 as compared to DC. Data are expressed as Mean + SD, n=5. NC: normal control; DC: diabetic control; PC: positive control; GM: galactomannan and CGA: chlorogenic acid

**Effect of chlorogenic acid and galactomannan on lipid profile:** in this study, both treatments improved more of the lipid profile, as summarised in Table 5. The concentration of triglyceride and LDL were significantly lowered (P < 0.001) in the GM and CGA groups, respectively. A comparable significant increment of HDL in the GM (0.04) and CGA (P-value of <0.001) was recorded. Low-density lipoprotein in GM and triglyceride in CGA were both significantly reduced at a level of 0.01. All in all, the lipid profile was improved by both CGA and GM.

**Table 5:** lipid profile among streptozotocin-induced diabetic mice

Groups	LDL [mg/dl]	HDL [mg/dl]	Triglyceride [mg/dl]	Cholesterol [mg/dl]
**NC**	***25.9 ±1.72	100± 39.1	*76.8 ±10.70	29.6 ± 4.63
**DC**	61.7 ± 10	40.9 ± 5.20	119 ±3.90	83.6 ± 5.20
**PC**	***27 ±2.82	*121 ± 5.75	*59.7 ± 27.50	***38.9 ± 6.55
**GM**	**39.3 **±** 11.4	*117 ±29.8 [0.04]	***55 **±** 28.50	***40.1 ± 9.17
**CGA**	***28.8± 5.20	*135 ± 34.90	*67.4 ± 7.46	***36.2 ± 4.04

Significance codes 0 ‘***’ 0.001 ‘**’ 0.01 ‘*’ 0.05 ‘*. **Note**: the mean difference is significant at p<0.05 as compared to DC. Data are expressed as Mean + SD, n=5 NC: normal control: DC: diabetic control, PC: positive control; GM: galactomannan and CGA: chlorogenic acid

## Discussion

Streptozotocin is a nitrosurea compound produced by *Streptomyces achromogenes*, which specifically induces DNA (deoxyribonucleic acid) strand breakage in beta cells causing diabetes mellitus. This leads to insulin deficiency which in turn increase the blood glucose level. Our research findings have gathered strong support from a multitude of esteemed scholars regarding the remarkable antidiabetic properties of both GM and CGA. The consensus among these well-known researchers underscores the strength and significance of GM's ability to effectively regulate blood glucose levels and CGA's potent antidiabetic effects. Galactomannan reverses diabetic-induced blood glucose levels. In type 2 diabetes mice, GM reduced lipids and blood sugar levels [31]. Further, it enhances the capacity for glycemic control (P < 0.05) [21]. It assists by helping the body reduce fasting blood glucose levels. It is proven in a blood sample tested for FBG associated with other factors [32]. Also, GM displays several promising properties and attributes for future applications as a therapeutic agent in anti-diabetic and anti-hyperlipidemic applications [33]. Considering this study (FBG, mean diff -176.8 (0.001)) and previous findings, it is revealed that the compound GM can be credited as a potential medication candidate for type 2 diabetes [18]. Green coffee supplementation reduces FBS [34]. Extracts of green coffee showed an equivalent outcome to this study on FBG [11]. The effect of CGA on FBG could be due to its role in pancreatic beta cell function [35], delaying starch breakdown [36], and the thermogenic, antioxidant, and anti-inflammatory properties of coffee [37]. Added to the reduction of FBG, this polyphenol helps to slow down postprandial blood sugar levels [36].

This study ascertained that kidney function tests improved by CGA and GM at a significant level of (p-value of <0.001). Chlorogenic acid improved, but GM significantly enhanced kidney function in comparison to DC. The treatments significantly enhanced liver and kidney function biomarkers in STZ-induced mice as compared to the diabetic control group, with varying degrees of significance. The previous studies on the effects of these treatments are stated as follows: CGA improves liver function [38]. The protection provided by CGA against liver function may be a result of its antioxidative and anti-inflammatory properties [39]. For a similar reason, GM reverses diabetic-induced impaired liver function [30]. It serves as a possible therapeutic option for such hepatic problems [19]. In a clinical trial conducted for 24 weeks, it was found that drinking a high dose of coffee (4 cups per day) was linked to a slight reduction in creatinine concentrations by -21.2% (0.001) [40]. Likewise, by reducing the levels of urea and creatinine in plasma, fenugreek galactomannan was able to inhibit diabetes-related kidney damage [33]. There also supporting research on the lipid profile. Green coffee supplementation reduces triglycerides while increasing HDL. Whereas, there was no statistically significant improvement in LDL [34]. In hypercholesterolemic rats treated with CGA, lipid depositions in the liver were dramatically reduced [41]. Galactomannan is found to be helpful in managing lipid profiles. Among these, serum levels of TC, LDL, and TG were significantly reduced and HDL significantly increased in GM-administered groups compared with the control group (P<0.05) [32]. *Trigonella foenum-graecum* seed powder solution had pronounced effects on improving lipid metabolism [25].

## Conclusion

In the present study, chlorogenic acid and galactomannan extracts from *Coffea arabica L* (green coffee) and *Trigonella foenum-graecum* L (fenugreek) seeds have shown a significant antihyperglycemic effect in STZ-induced diabetic mice. Administration of both extracts was found to improve lipid profiles (increased HDL, reduced LDL, TG, and total cholesterol) as well as liver and kidney functions. Both extracts exhibit comparable antidiabetic effects. Therefore, CGA and GM may be used to stabilize fasting blood glucose and prevent diabetes-induced associated factors.

### What is known about this topic

- There have been conflicting findings on the effect of chlorogenic acid-treated and galactomannan-treated on fasting blood glucose;
- Limited studies on the effects of chlorogenic acid-treated and galactomannan-treated extracts on glucose tolerance;
- Diabetes affects lipid profile, liver and kidney functions.

### What this study adds

- Both chlorogenic acid-treated and galactomannan-treated extracts from Coffea arabica L and Trigonella foenum-graecum L seeds respectively, reduce hyperglycemia;
- There is a comparable effect of chlorogenic acid-treated and galactomannan-treated on glucose tolerance;
- Lipid profile, kidney and liver functions are improved following chlorogenic acid-treated and galactomannan-treated treatments.
